# A comprehensive study of the association between drug hepatotoxicity and daily dose, liver metabolism, and lipophilicity using 975 oral medications

**DOI:** 10.18632/oncotarget.4400

**Published:** 2015-06-24

**Authors:** Zuquan Weng, Kejian Wang, Haibo Li, Qiang Shi

**Affiliations:** ^1^ Division of Systems Biology, National Center for Toxicological Research, Food and Drug Administration, Jefferson, AR 72079, USA; ^2^ Department of Clinical Laboratory Medicine, Nantong Maternal and Child Health Hospital, Nantong, Jiangsu, 226018, China

**Keywords:** Pathology Section, hepatotoxicity, metabolism, lipophilicity, dose, oral

## Abstract

It was recently suggested that daily dose, liver metabolism and lipophilicity were associated with an oral drug's potential to cause hepatotoxicity, but this has not been widely accepted. A likely reason is that published data lack comprehensiveness, as they were based on only about one third of all FDA approved single-active-ingredient oral prescription drugs. Here the 975 oral drugs used worldwide which have a Defined Daily Dose (DDD) designated in the World Health Organization's Anatomical Therapeutic Chemical classification system and whose hADRs potential and metabolism data are available in the Micromedex Drugdex^®^ compendium were studied, with their lipophilicity calculated by the partition coefficient LogP. Of the 975 drugs examined, 49% (478) have the potential to induce at least one type of hepatic adverse drug reactions (hADRs) such as fatal hepatotoxicity, acute liver failure, significant ALT/AST elevation, hepatitis, and jaundice. By single factor analysis, a higher DDD (≥100 mg) was found to be associated with all types of hADRs, and extensive liver metabolism (≥50%) was associated with a subset of hADRs including fatal hADRs, hepatitis and jaundice, while LogP showed no relation to any types of hADRs. Contrary to previous reports, none of the combination, neither DDD and liver metabolism, nor DDD and LogP, was found to be more predictive of hADRs than using DDD or liver metabolism alone. These data provide convincing evidence that a higher daily dose and extensive liver metabolism, but not lipophilicity, are independent but not synergistic risk factors for oral drugs to induce hepatotoxicity.

## INTRODUCTION

Drug hepatotoxicity is the leading cause of acute liver failure and unfavorable regulatory actions such as drug non-approvals or market withdrawals. Tremendous efforts have been undertaken to predict which drug is more likely than the others to induce hepatic adverse drug reactions (hADRs), but relatively little progress has been made [1]. Inspired by the observation that drugs received unfavorable regulatory actions due to liver injury are very often administrated at high doses [2, 3], a recent study examined 230 mostly prescribed U.S. drugs and concluded that a higher dose (that is, ≥50 mg) is a risk factor for severe hADRs induced by oral medications [4]. Using a slightly smaller number of 207 drugs, the same group later on reported that extensive liver metabolism (that is, ≥50% liver metabolism) is another risk factor for an oral drug to induce hADRs, and the combination of daily dose and liver metabolism appears to be synergistic in predicting liver risks [5]. More recently, based on data from 164 drugs, another group reported that the combination of oral dose and lipophilicity was remarkably more effective than either alone in predicting a drug's hADR potential [6]. However, this combination appeared ineffective in a larger number of 254 drugs [7]. The general belief remains that none of these medication characteristics are able to predict hADRs with high confidence [1]. An outstanding caveat is that all previous studies lack comprehensiveness, that is, the number of drugs examined is relatively small as compared to all FDA approved new molecular entities (NMEs) or currently used oral prescription drugs.

According to a recent report, the FDA has approved 1,453 new molecular entities from the year 1827 to 2013 [8]. However, this is an underestimate by about 10%. According to the FDA official statistics, 1,527 NMEs were approved by the FDA during the period of 1940 to 2011 (http://www.fda.gov/AboutFDA/WhatWeDo/History/ProductRegulation/SummaryofNDAApprovalsReceipts1938tothepresent/default.htm). In the year 2012 to 2014, the FDA has approved an additional 107 NMEs (http://www.fda.gov/Drugs/DevelopmentApprovalProcess/DrugInnovation/default.htm). Therefore at least a total of 1,634 NMEs has been approved by the FDA. Though some approved drugs were later on discontinued or withdrawn from the market [9, 10], the number of currently used single-active-ingredient oral prescription drugs, as downloaded in June 2014 from the FDA Online Label Repository using the FDALabel database [11], is 736. This raised the possibility that previous reports on dose, metabolism and lipophilicity affecting hADRs could be biased, as only a small fraction of drugs, that is, only around 15% of all the FDA approved NMEs or 34% of all currently used U.S. oral prescription drugs, were included for analysis. The present study aimed at to scrutinize the role of dose, metabolism and lipophilicity, each alone or in combination, in predicting an oral drug's hADR potential using a comprehensive list of drugs covering nearly all human oral medications.

## RESULTS

As of July 2013, the WHO ATC/DDD system contains 5,015 drug records (Supplementary Table 1), with 2,338 drugs having DDDs assigned (Supplementary Table 2), among which 1,013 drugs are used only by oral administration and 379 drugs are used both orally and non-orally (Supplementary Table 3). Of the 1,392 drugs that can be used by oral route, 975 drugs (Supplementary Table 4) were found to be included in the Micromedex Drugdex^®^ compendium and therefore were chosen for further analysis.

Of the 975 drugs analyzed, 478 (49%) drugs were found to be associated with at least one type of hADRs. Specifically, 150, 208, 37, 309, 251, 61, and 291 drugs were shown to have the potential to induce fatal hADRs, liver failure, liver transplantation, jaundice, biomarker increase, hepatomegaly and hepatitis, respectively (Supplementary Table 4). Interestingly, though a previous report using 230 mostly prescribed U.S. drugs found no drugs causing liver transplantation when their daily doses were equal or less than 10 mg [4], here we found that ramipril with a DDD of 2.5 mg is associated with liver transplantation.

The Cochran-Armitage trend test was used to examine if DDDs or LogP are associated with a drug's hADR potential. Table 1 shows that the percentage of drugs inducing all major types of hADRs was significantly increased when DDDs became higher. Specifically, among the 169 drugs whose DDDs are less than 10 mg, only 12 drugs (7%) can cause fatal hADRs. However, among the 288 drugs whose DDDs are in the range of 10 to 100 mg, 35 drugs (12%) can induce fatal hADRs. As for the 518 drugs whose DDDs are equal or large than 100 mg, 103 drugs (20%) can trigger fatal hADRs. The difference among dose groups was statistically significant (*p* < 0.001). Similar results were obtained regarding other types of hADRs including liver failure, liver transplantation, jaundice, biomarker increase, hepatomegaly, and hepatitis, either individually or combined together. In contrast, Table 2 shows the percentage of drugs inducing different types of hADRs (except for jaundice) was not associated with LogP (*p* > 0.05).

**Table 1:** Association between Defined Daily Dose and hepatic adverse drug reactions

DDD Group (Drug Number)	DDD < 10 mg (*n* = 169)	10 ≤ DDD < 100 mg (*n* = 288)	DDD ≥ 100 mg (*n* = 518)	*P*-value
hADRs Type				
Fatal hADRs	12 (7%)	35 (12%)	103 (20%)	1.31E-05^***^
Liver Failure	13 (8%)	58 (20%)	137 (26%)	3.42E-07^***^
Liver Transplantation	1 (1%)	8 (3%)	28 (5%)	2.41E-03^**^
Jaundice	36 (21%)	83 (29%)	190 (37%)	8.43E-05^***^
Biomarker Increase	23 (14%)	67 (23%)	161 (31%)	3.43E-06^***^
Hepatomegaly	6 (4%)	12 (4%)	43 (8%)	8.29E-03^**^
Hepatitis	27 (16%)	74 (26%)	190 (37%)	5.94E-08^***^
Severe hADRs	16 (9%)	60 (21%)	149 (29%)	1.53E-07^***^
All hADRs	60 (36%)	130 (45%)	288 (56%)	1.60E-06^***^

*P* value was determined using the Cochran-Armitage test.

* *P* < 0.05

** *P* < 0.01

*** *P* < 0.001

**Table 2:** Association between LogP and hepatic adverse drug reactions

LogP Group (Drug Number)	LogP < 1(*n* = 214)	1 ≤ LogP < 3(*n* = 378)	LogP ≥ 3(*n* = 360)	*P*-value
hADR Type				
Fatal hADRs	35 (16%)	46 (12%)	68 (19%)	2.35E-01
Liver Failure	55 (26%)	64 (17%)	88 (24%)	8.97E-01
Liver Transplantation	9 (4%)	8 (2%)	20 (6%)	2.41E-01
Jaundice	69 (32%)	98 (26%)	141 (39%)	2.43E-02^*^
Biomarker Increase	54 (25%)	94 (25%)	101 (28%)	3.94E-01
Hepatomegaly	19 (9%)	23 (6%)	19 (5%)	1.04E-01
Hepatitis	68 (32%)	107 (28%)	115 (32%)	8.15E-01
Severe hADRs	58 (27%)	72 (19%)	94 (26%)	8.69E-01
All hADRs	105 (49%)	177 (47%)	192 (53%)	2.23E-01

*P* value was determined using the Cochran-Armitage test.

* *P* < 0.05

** *P* < 0.01

*** *P* < 0.001

The Fisher's exact test was used to determine if extensive liver metabolism, that is, ≥50% of liver metabolism as defined in a previous report [5], is associated with a drug's hADR potential. As shown in Table 3, among the 483 drugs that are subjected to extensive liver metabolism, 99 drugs (20%) can cause fatal hADRs, while only 18 out of 162 (11%) drugs that do not undergo extensive liver metabolism can induce fatal hADRs. Such difference was statistically significant (*p* < 0.01). Similarly, extensive liver metabolism was also associated with jaundice, but not the remaining individual types of hADRs. Nevertheless, the pooled hADRs were indeed significantly associated with extensive liver metabolism (*p* < 0.01). In contrast to the data in Table 1, none of the statistically significant association reached the confidence level of *p* < 0.001 in Table 3.

**Table 3:** Association between liver metabolism and hepatic adverse drug reactions

Metabolism Group (Drug Number) hADRs Type	Liver Metabolism ≥ 50% (*n* = 483)	Liver Metabolism < 50% (*n* = 162)	*P*-value
hADRs Type			
Fatal hADRs	99 (20%)	18 (11%)	3.98E-03^**^
Liver Failure	124 (26%)	36 (22%)	2.20E-01
Liver Transplantation	20 (4%)	6 (4%)	5.09E-01
Jaundice	195 (40%)	44 (27%)	1.54E-03^**^
Biomarker Increase	164 (34%)	40 (25%)	1.70E-02
Hepatomegaly	33 (7%)	13 (8%)	7.58E-01
Hepatitis	167 (35%)	41 (25%)	1.75E-02^*^
Severe hADRs	135 (28%)	37 (23%)	1.20E-01
All hADRs	277 (57%)	73 (45%)	4.36E-03^**^

*P* value was determined using the Fisher's exact test.

* *P* < 0.05

** *P* < 0.01

*** *P* < 0.001

We next examined if the combination of DDD, metabolism or LogP would enhance the prediction power as previously suggested [5, 6]. The overall results comparing the performance of DDD alone to its combination with either LogP or metabolism were presented in Table 4. For all the individual types of hADRs except hepatomegaly, the combination of DDD and LogP produced a slightly higher crude odds ratio than using DDD alone. However, none of such increase exceeded 43% of the odds ratio associated with DDD alone, and these slight increases were accompanied by a sharp decrease in the true positive rate (TPR), with all of which becoming less than 50%. It is apparent that the combination of DDD and LogP produced a negligible increase of the crude odds ratio at the expense of sacrificing the TPR significantly. Similarly, the combination of DDD and liver metabolism produced even worse prediction power, as the crude odds ratio was actually decreased for several types of hADRs such as liver failure and liver transplantation, and again the TPR was markedly reduced for all types of hADRs, with the majority of them decreased to less than 50%. Table 4 clearly demonstrates that none of the combination, neither was it DDD and metabolism, nor DDD and LogP, provided added value in predicting a drug's hADR potential than using DDD alone.

**Table 4:** Predicting hepatic adverse drug reactions using the combination of Defined Daily Dose, liver metabolism or LogP

	Criteria		hADRs		OR (95% CI)	TPR	FPR
			Y	N			
Fatal hADRs	DDD ≥ 100 mg	Y	103	415	2.17^***^ (1.47–3.21)	69%	50%
		N	47	410			
	DDD ≥ 100 mg & LogP ≥ 3	Y	44	117	2.46^***^ (1.60–3.73)	30%	15%
		N	105	686			
	DDD ≥ 100 mg & LM ≥ 50%	Y	66	171	2.70^***^ (1.76–4.15)	56%	32%
		N	51	357			
Liver Failure	DDD ≥ 100 mg	Y	137	381	1.95^***^ (1.40–2.73)	66%	50%
		N	71	386			
	DDD ≥ 100 mg & LogP ≥ 3	Y	55	106	2.18^***^ (1.47–3.20)	27%	14%
		N	152	639			
	DDD ≥ 100 mg & LM ≥ 50%	Y	75	162	1.76^**^ (1.20–2.57)	47%	33%
		N	85	323			
Liver Transplantation	DDD ≥ 100 mg	Y	28	490	2.84^**^ (1.28–6.92)	76%	52%
		N	9	448			
	DDD ≥ 100 mg & LogP ≥ 3	Y	15	146	3.59^***^ (1.69–7.43)	41%	16%
		N	22	769			
	DDD ≥ 100 mg & LM ≥ 50%	Y	13	224	1.76 (0.74–4.20)	50%	36%
		N	13	395			
Jaundice	DDD ≥ 100 mg	Y	190	328	1.65^***^ (1.24–2.19)	61%	49%
		N	119	338			
	DDD ≥ 100 mg & LogP ≥ 3	Y	79	82	2.36^***^ (1.65–3.39)	26%	13%
		N	229	562			
	DDD ≥ 100 mg & LM ≥ 50%	Y	117	120	2.29^***^ (1.62–3.23)	49%	30%
		N	122	286			
Biomarker Increase	DDD ≥ 100 mg	Y	161	357	1.84^***^ (1.35–2.50)	64%	49%
		N	90	367			
	DDD ≥ 100 mg & LogP ≥ 3	Y	61	100	1.96^***^ (1.34–2.84)	24%	14%
		N	188	603			
	DDD ≥ 100 mg & LM ≥ 50%	Y	98	139	2.01^***^ (1.41–2.86)	48%	32%
		N	106	302			
Hepatomegaly	DDD ≥ 100 mg	Y	43	475	2.21^**^ (1.22–4.13)	70%	52%
		N	18	439			
	DDD ≥ 100 mg & LogP ≥ 3	Y	16	145	1.83 (0.94–3.41)	26%	16%
		N	45	746			
	DDD ≥ 100 mg & LM ≥ 50%	Y	23	214	1.80 (0.94–3.44)	50%	36%
		N	23	385			
Hepatitis	DDD ≥ 100 mg	Y	190	328	2.04^***^ (1.52–2.74)	65%	48%
		N	101	356			
	DDD ≥ 100 mg & LogP ≥ 3	Y	71	90	2.06^***^ (1.43–2.96)	24%	14%
		N	219	572			
	DDD ≥ 100 mg & LM ≥ 50%	Y	108	129	2.58^***^ (1.81–3.68)	52%	30%
		N	100	308			
Severe hADRs	DDD ≥ 100 mg	Y	149	369	2.02^***^ (1.47–2.80)	66%	49%
		N	76	381			
	DDD ≥ 100 mg & LogP ≥ 3	Y	57	104	2.05^***^ (1.39–2.99)	25%	14%
		N	167	624			
	DDD ≥ 100 mg & LM ≥ 50%	Y	83	154	1.93^***^ (1.33–2.80)	48%	33%
		N	89	319			
All hADRs	DDD ≥ 100 mg	Y	288	230	1.76^***^ (1.35–2.29)	60%	46%
		N	190	267			
	DDD ≥ 100 mg & LogP ≥ 3	Y	102	59	1.95^***^ (1.35–2.81)	22%	12%
		N	372	419			
	DDD ≥ 100 mg & LM ≥ 50%	Y	157	80	2.19^***^ (1.55–3.09)	45%	27%
		N	193	215			

*P* value was determined using the Fisher's exact test. Y, positive; N, negative; DDD, Defined Daily Dose; OR, odds ratio; CI, confidence interval; LM, liver metabolism; TPR, true positive rate; FPR, false positive rate.

* *P* < 0.05

** *P* < 0.01

*** *P* < 0.001

The comparison between using liver metabolism alone and its combination with LogP or DDD, as well as those using LogP alone and its combination with liver metabolism or DDD, also showed a similar tendency, that is, no added value was obtained when combining two factors, as compared to using either alone. Detailed results were presented in the Supplementary Table 5.

To further clarify the possible interactions among DDD, liver metabolism and LogP, the multivariate logistic regression analysis was performed. As shown in Table 5, no statistically significant interactions between DDD and LogP, or DDD and metabolism, were observed in predicting all the hADRs types examined (*p* > 0.01). Similar results were obtained in analyzing the possible interaction between LogP and metabolism (data not shown). These results demonstrate that DDD, live metabolism, and LogP do not interact with each other in predicting a drug's hADR potential.

**Table 5:** Interactions between oral dose and LogP, or oral dose and hepatic metabolism analyzed by logistic regression

	Oral Dose × LogP			Oral Dose × Hepatic Metabolism		
	Adjusted OR	95% CI	*P*-value	Adjusted OR	95% CI	*P*-value
Fatal hADRs	1.31	0.62–2.77	0.484	2.14	0.69–6.66	0.190
Liver Failure	1.44	0.75–2.78	0.270	1.12	0.45–2.8	0.800
Liver Transplantation	1.58	0.34–7.34	0.557	0.00	0.00–+∞	0.986
Jaundice	1.26	0.72–2.23	0.419	1.19	0.51–2.77	0.687
Biomarker Increase	1.44	0.78–2.66	0.240	1.32	0.56–3.1	0.532
Hepatomegaly	5.17	1.26–21.22	0.022^*^	2.79	0.69–11.25	0.149
Hepatitis	1.48	0.82–2.67	0.188	2.68	1.17–6.16	0.020^*^
Severe hADRs	1.17	0.62–2.22	0.627	1.16	0.47–2.86	0.755
All hADRs	1.15	0.67–1.97	0.610	1.40	0.66–2.95	0.382

The Wald test was used to determine the statistical significance of interacting variable in logistic regression. OR, odds ratio; CI, confidence interval.

* *P* < 0.05

** *P* < 0.01

*** *P* < 0.001

As previous reports used either 50 mg [4] or 100 mg [6] as a cutoff for daily dose, additional analysis was performed using 50 mg as a cutoff to facilitate a more direct comparison between our dataset and those published. Nearly identical results were obtained in these additional analyses (data not shown).

## DISCUSSION

This is the most comprehensive study so far that aimed at examining the relation between a drug's hADR potential and its daily dose, liver metabolism, or lipophilicity. Our results not only consolidated and but also remarkably expanded the view that a higher daily dose is a risk factor for oral drugs to induce hADRs. Though a previous report based on 230 drugs failed to demonstrate a statistically significant association between daily dose and ALT > 3 × ULN, nor daily dose and jaundice [4], here these associations became clearly established by using a more than 4-fold larger number of drugs. Additionally, daily dose was found to be strongly associated with hepatitis and hepatomegaly, two types of hADRs that were not examined in the previous study [4]. This finding is of particular significance because nearly one third (291 out of 975) of drugs were found to be associated with hepatitis. Our data provide convincing evidence that a higher daily dose is a risk factor for all major types of hADRs induced by oral medications.

In partial agreement with a previous study [5], we found that only a subset of hADRs was associated with extensive liver metabolism. It is notable that both our study and the previous report [5] showed that fatal DILI and ALT > 3 × ULN, but not liver transplantation, were related to extensive liver metabolism. A notable difference between two studies is that jaundice, but not liver failure, was shown to be related to extensive liver metabolism in our dataset, but the opposite was observed in the previous study [5]. We additionally found that hepatitis was also significantly associated with extensive liver metabolism. These consistencies and discrepancies are most likely due to the number of drugs studied. Regardless of the differences, both the previous report [5] and our present study clearly show that extensive liver metabolism was associated with only some but not all types of hADRs, indicating that liver metabolism is a less important risk factor than daily dose for predicting an oral drug's hADR potential. Further supporting this notion, a recent report [7] based on 254 drugs showed that higher daily doses were more effective than cytochrome P450s (CYP) mediated metabolism in predicting a drug's hADRs potential.

Contrary to the common assumption [12] and a recent report [5], though DDD and liver metabolism each alone contributes to a drug's hADR potential, their combination does not have a synergistic effect. This is likely because that the qualitative but not quantitative nature of liver metabolism plays a more important role in triggering hADRs. Regardless of the reasons, our data clearly show that a previous report about the enhanced power of the combination of daily dose and liver metabolism in predicting hADRs [5] needs to be revisited. Of note, our data are in line with a more recent report which showed that, among 254 oral drugs, though “drugs that are cytochrome P450s enzymes substrates” were more likely to induced hADRs than those that are not substrates, this tendency is dose-independent [7].

Another interesting finding is that when lipophilicity was combined with DDD, no added value was obtained. This is in contrast to a recent report which showed that a combination of LogP ≥ 3 and DDD ≥ 100 mg was remarkable more predictive of a drug's hADR potential than using either criterion alone [6], but is consistent with a more recent publication that was based on a slightly large number of drugs [7]. It seems apparent that lipophilicity, either alone or in combination with other factors, does not play a significant role in predicting an oral drug's liver risks.

Our study provides a unique quantitative estimate of the hADR risks in overall human medication use, that is, 49% of the drugs examined have the potential to induce at least one type of hADRs. This result may help health care providers, regulators and pharmaceutical industries better evaluate the risks of hADRs associated with drugs.

Due to the relatively rareness of severe hADRs and ethical issues, many theories are difficult to be tested experimentally and are therefore highly speculative at this time, and controversies are not uncommon in this field [13]. Given that few general rules have been established in the past decades, any new rules such as the one dubbed “rule of 2” [6] shall be treated with caution. Similar caution and scrutiny should be exercised in viewing the recently developed new experimental approaches aimed at predicting hADRs, such as those using high-content analysis of cellular injury [14], mitochondrial dysfunctions [15] and a combination of mechanistic toxicity endpoints [16], as all these studies were also based upon drugs in the low hundreds. To minimize further confusions, our study highlights the importance of avoiding overgeneralization before comprehensive studies are in place. By the same token, the present study has two apparent limitations. First, the drug list does not cover all drugs used by human kind. Secondly, understandings of a drug's hADRs potential, as well as its metabolism, are evolving with time and the present study represents only a snapshot of current knowledge deposited in a single database, the Micromedex Drugdex^®^ compendium. Our findings need to be further evaluated when more data become available and new drugs come to the market in the future.

## MATERIALS AND METHODS

All drug records in the World Health Organization's (WHO) Anatomical Therapeutic Chemical classification and Defined Daily Dose (ATC/DDD) system were manually collected from the official website at http://www.whocc.no/atc_ddd_index/ in July 2013.

Only drugs with an assigned DDD were selected for further analysis. Though previous reports [4, 6, 7] used other sources than the WHO ATC/DDD system to determine a drug's daily dose, we chose not to do so. This not only minimizes subjectivity but also reduces possible errors, as the WHO ATC/DDD system represents the consensus achieved among worldwide experts and has been considered as a reliable information source for nearly 30 years. Notably, the WHO ATC/DDD system also updates the DDDs when the dosage changes over time (http://www.whocc.no/filearchive/publications/2014_guidelines.pdf).

If a drug is used by both oral and non-oral routes of administration, it was also included in the present study, but only the oral DDD was used for analysis. For a small number of oral drugs that are used for multiple diseases and therefore have multiple DDDs, the average DDDs were used.

In line with previous reports [4, 5], the Micromedex Drugdex^®^ compendium was used to collect the drug metabolism data and to determine if a drug causes any types of hADRs. For data collection, the criteria used in previous reports [4, 5] were used with slight modifications. These modifications include (1) though previous reports focused on “alanine aminotransferase (ALT) greater than 3 times the upper limit of normal” (ALT > 3 × ULN) as one important type of hADRs [4, 5], we used a more general term, that is, biomarker increase, to cover this type of hADRs, for which we included not only ALT > 3 × ULN, but also other findings such as “clinically significant increase of ALT and/or aspartate aminotransferase (AST)”, or “ALT/AST elevations that led to drug withdrawal”, (2) we additionally collected drugs that are associated with hepatitis and/or hepatomegaly, as we noticed that lots of drugs can cause these types of hADRs, (3) we used the term “severe hADRs” to cover hADRs leading to death or causing acute liver failure or necessitating liver transplantation. Other criteria are the same as previous reports [4, 5]. As the Micromedex Drugdex^®^ compendium is updating constantly, and our data collection was completed in March 2014, updates after that will not be reflected in the present manuscript.

In line with a previous report, drug lipophilicity was determined by the partition coefficient LogP [6], which was calculated using the online software ALOGPS 2.1 (http://www.vcclab.org/lab/alogps/start.html).

Statistical analysis: For single factor analysis, the Cochran-Armitage test was used to determine the statistical significance of the association between DDDs and hADRs, and the Fisher's exact test was used for liver metabolism and hADRs. The possible interaction between DDD and LogP or liver metabolism, or LogP and liver metabolism, was determined by multivariate logistic regression model using the Wald test. A *p* value less than 0.05, 0.01 or 0.001 was considered as statistically meaningful.

## SUPPLEMENTARY TABLES LEGENDS













## Abbreviations

hADRs: hepatic adverse drug reactions
DDD: Defined Daily Dose
NMEs: new molecular entities
FDA: Food and Drug Administration
WHO: World Health Organization
ATC/DDD: Anatomical Therapeutic Chemical classification and Defined Daily Dose system
ALT: alanine aminotransferase
AST: aspartate aminotransferase
