# Supplementary material for: A comprehensive study of the association between drug hepatotoxicity and daily dose, liver metabolism, and lipophilicity using 975 oral medications
Source: Oncotarget. 2015 Jun 24;6(19):17031–8. doi: 10.18632/oncotarget.4400 (PMC4627289; doi:10.18632/oncotarget.4400)
Supplement: Supplementary file 1 [file oncotarget-06-17031-s001.pdf]

SUPPLEMENTARY TABLES LEGENDS

Supplementary Table S1. All 5,015 drug records in the WHO ATC/DDD system

Supplementary Table S2. The 2,338 drugs with assigned DDDs

Supplementary Table S3. The 1,392 drugs that can be used orally

Supplementary Table S4. The 975 drugs analyzed in the present study

Supplementary Table S5. Predicting hepatic adverse drug reactions using the combination of Defined Daily Dose, liver metabolism or LogP
